# Pulmonary Vein Stenosis Mimicking Nonspecific Interstitial Pneumonia

**DOI:** 10.1155/2015/290391

**Published:** 2015-12-08

**Authors:** Karthika R. Linga, Andras Khoor, Jonathan A. Phelan, Isabel Mira-Avendano

**Affiliations:** ^1^Department of Pulmonary Medicine, Mayo Clinic, 4500 San Pablo Road, Jacksonville, FL 32224, USA; ^2^Department of Critical Care, Mayo Clinic, 4500 San Pablo Road, Jacksonville, FL 32224, USA; ^3^Department of Laboratory Medicine and Pathology, Mayo Clinic, 4500 San Pablo Road, Jacksonville, FL 32224, USA; ^4^Department of Radiology, Mayo Clinic, 4500 San Pablo Road, Jacksonville, FL 32224, USA

## Abstract

Pulmonary vein stenosis (PVS) is a known complication after catheter ablation of arrhythmias. Surprisingly, little information is available on its manifestations in the lung. We describe the case of a 39-year-old woman who presented from an outside hospital with worsening shortness of breath after catheter ablation of pulmonary veins for atrial fibrillation. After an initial diagnosis of pneumonia and its nonimprovement with antibiotics, a surgical lung biopsy was done and interpreted as nonspecific interstitial pneumonia (NSIP) with vascular changes consistent with pulmonary arterial hypertension. Later, she was admitted to our institution where a transthoracic echocardiogram (TTE) and subsequent computed tomography (CT) angiogram of the heart showed severe stenosis of all four pulmonary veins. The previous lung biopsy was rereviewed and reinterpreted as severe parenchymal congestion mimicking NSIP. Our case demonstrates that PVS is an underrecognized complication of catheter ablation, and increased awareness among both clinicians and pathologists is necessary to avoid misdiagnosis.

## 1. Case Report

**Figure 1:**
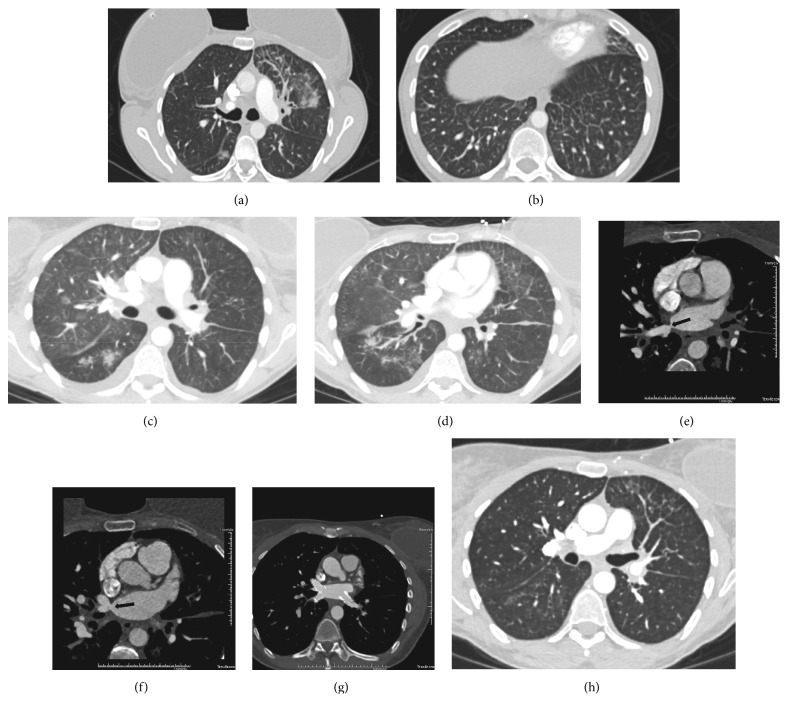
(a) and (b) Axial computed tomography (CT) images of prepulmonary vein stenting demonstrate patchy opacities and ground-glass and septal thickening reflecting manifestations of pulmonary vein stenosis with congestion and edema. Opacities are most pronounced in the left upper lobe secondary to complete occlusion of the superior left pulmonary vein ostium. (c) and (d) Follow-up axial CT images of prepulmonary vein stenting demonstrate progression of patchy opacities and ground-glass compatible with increased pulmonary venous congestion and edema. Additionally, small pleural effusions are now visible. (e) and (f) Cardiac CT shows narrowing of the right superior and inferior pulmonary vein ostia (arrows). Although not shown, narrowing of the left inferior ostium and complete occlusion of the left superior pulmonary vein ostium was noted. (g) Contrast-enhanced CT of postpulmonary vein stenting demonstrates marked improvement of pulmonary vein narrowing. The superior left pulmonary vein ostium was not stented and remained occluded. (h) Axial chest CT in lung windows shows substantial improvement of ground-glass and septal thickening. Findings remain the most pronounced in the left upper lobe, as the superior left pulmonary vein ostium was not stented.

A 39-year-old woman with no history of smoking presented with a nine-month history of progressive dyspnea on exertion and dry cough. One month before the onset of symptoms, she underwent a successful cardiac ablation of pulmonary veins for refractory atrial fibrillation. Computed tomography (CT) of the chest was done eight weeks prior to presentation and revealed bilateral scattered ground-glass opacities, diffuse septal thickening, and patchy consolidations in left lung (Figures 1(a) and 1(b)). After failed conservative therapy with antibiotics, diuresis, and a nondiagnostic bronchoscopy, she underwent video-assisted thoracoscopic surgery (VATS) with a left, lower-lobe wedge biopsy. Pathology identified diffuse pulmonary fibrosis associated with advanced arteriopathic changes of pulmonary hypertension without any sparing/alternating of the normal lung, which led to a diagnosis of NSIP. Despite lacking any systemic symptoms of connective tissue disease (CTD), she was started on high-dose steroids based on a tissue diagnosis of NSIP. Her symptoms continued to worsen in spite of six weeks of the above treatment, and she eventually presented to our hospital.

**Figure 2:**
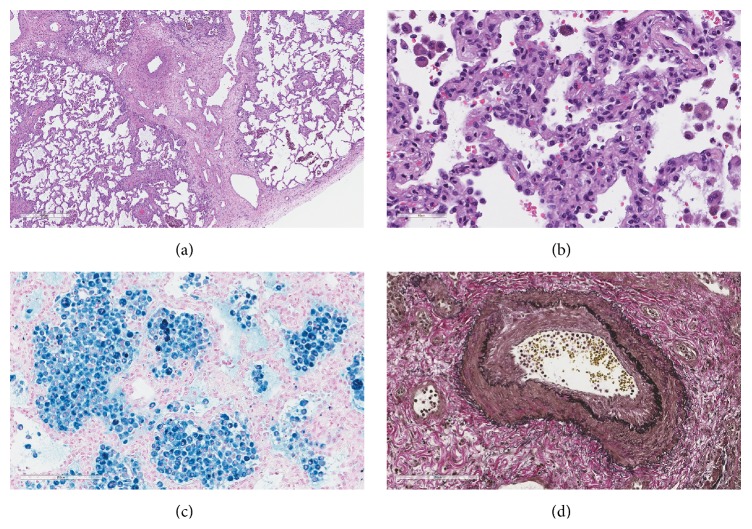
Lung biopsy from the patient with pulmonary vein stenosis. (a) Low magnification shows thickening of the interlobular septa (hematoxylin and eosin stain, original magnification ×4). (b) High magnification exhibits nonspecific alveolar septal thickening (hematoxylin and eosin stain, original magnification ×40). (c) Prussian blue stain reveals intra-alveolar hemosiderin-filled macrophages (original magnification ×20). (d) Verhoeff-Van Gieson stain highlights medial hypertrophy and intimal proliferation in a muscular pulmonary artery (original magnification ×20).

At admission, she was comfortable at rest, saturating at 92% with clear lungs on auscultation. Repeat imaging showed continued presence of diffuse septal thickening and ground-glass opacities (Figures 1(c) and 1(d)). Because of the temporal relation of her symptoms to the catheter ablation, nonimprovement despite steroid therapy, and negative CTD serologies, we further expanded our search for an alternative diagnosis. A TTE showed normal mPAP of 23 mm Hg with normal size and function of the right ventricle and a severely elevated right upper pulmonary vein velocity of > 103 cm/second, suggestive of pulmonary venous stenosis. This was further confirmed by a CT angiogram of heart, which showed severe stenosis of all four pulmonary veins (Figures 1(e) and 1(f)). The previous lung biopsy was rereviewed and showed changes consistent with severe congestion, including thickening of the interlobular and alveolar septa and accumulation of hemosiderin-laden macrophages in the alveoli (Figures 2(a) and 2(b)). An iron stain confirmed the latter finding (Figure 2(c)). Although the alveolar septal thickening was somewhat reminiscent of nonspecific interstitial pneumonia, it was thought to be secondary to congestion and, therefore, the term “nonspecific interstitial pneumonia” was deemed inappropriate. Similarly, the pulmonary arterial changes could be explained by the upstream effect of pulmonary venous outflow impairment (Figure 2(d)).

Subsequently, her steroids were tapered, and, after we discussed the case with cardiologists at our institution, the patient underwent balloon angioplasty with pulmonary vein stenting (Figure 1(g)). Six weeks later, she demonstrated significant clinical and radiographic improvement (Figure 1(h)), with an isolated mild reduction in her diffusion capacity on pulmonary function testing.

## 2. Discussion

The incidence of PVS after catheter ablation for atrial fibrillation ranges from 1% to 3% [1]. The major symptoms of PVS include cough, hemoptysis, dyspnea, and chest pain, but these depend on the severity and the time course of the process [2]. Lung parenchymal abnormalities are indirect evidence of PVS and manifest radiographically as multifocal opacities, nodular lesions, unilateral effusions, and interstitial septal thickening. These can lead to erroneous diagnoses of airway disease, pneumonia, bronchopulmonary malignancies, and pulmonary embolism, and patients are therefore unnecessarily subjected to antibiotics, anticoagulants, and in some cases even partial pneumonectomies [3, 4]. Histologically, PVS manifests as a fibromuscular proliferation with occlusion of pulmonary veins. The pulmonary parenchyma is congested with interlobular and alveolar septal thickening, arteriopathic changes, and, most importantly, hemosiderosis. Similar pulmonary parenchymal changes can be seen with other postcapillary pulmonary hypertension etiologies including pulmonary venoocclusive disease, which can be differentiated from PVS and mitral stenosis by the presence of characteristic thromboembolic changes in medium-sized veins. Severely congested lungs can be separated from NSIP by the presence of hemosiderin-laden macrophages and a corroborating clinical history.

In conclusion, postablative patients with any pulmonary symptoms should have a high suspicion for PVS. It not only mimics other disease entities clinically and radiographically, but also can mimic other etiologies histologically, as seen with our case. Such delay and misdiagnoses subject patients to erroneous treatment and can cause irreversible secondary pulmonary parenchymal and vasculature changes with poorer outcomes [5].

## References

[1] Holmes D. R., Monahan K. H., Packer D. (2009). Pulmonary vein stenosis complicating ablation for atrial fibrillation: clinical spectrum and interventional considerations. *JACC Cardiovascular Interventions*.

[2] Saad E. B., Marrouche N. F., Saad C. P. (2003). Pulmonary vein stenosis after catheter ablation of atrial fibrillation: emergence of a new clinical syndrome. *Annals of Internal Medicine*.

[3] Lee J. Y., Chon G. R., Park J. H., Kang B. J., Shim T. S., Jo K.-W. (2015). Massive hemoptysis due to pulmonary vein stenosis following catheter ablation for atrial fibrillation. *Respiratory Care*.

[4] Yang H.-M., Lai C. K., Patel J. (2007). Irreversible intrapulmonary vascular changes after pulmonary vein stenosis complicating catheter ablation for atrial fibrillation. *Cardiovascular Pathology*.

[5] Prieto L. R., Schoenhagen P., Arruda M. J., Natale A., Worley S. E. (2008). Comparison of stent versus balloon angioplasty for pulmonary vein stenosis complicating pulmonary vein isolation. *Journal of Cardiovascular Electrophysiology*.

